# Mouse models and anti-HER2 therapies

**DOI:** 10.18632/oncotarget.1481

**Published:** 2013-11-15

**Authors:** Ariella B. Hanker, Rebecca S. Cook, Carlos L. Arteaga

**Affiliations:** Departments of Medicine and Cancer Biology, Breast Cancer Research Program, Vanderbilt-Ingram Cancer Center, Vanderbilt University, Nashville, TN, USA

**Figure 1:**
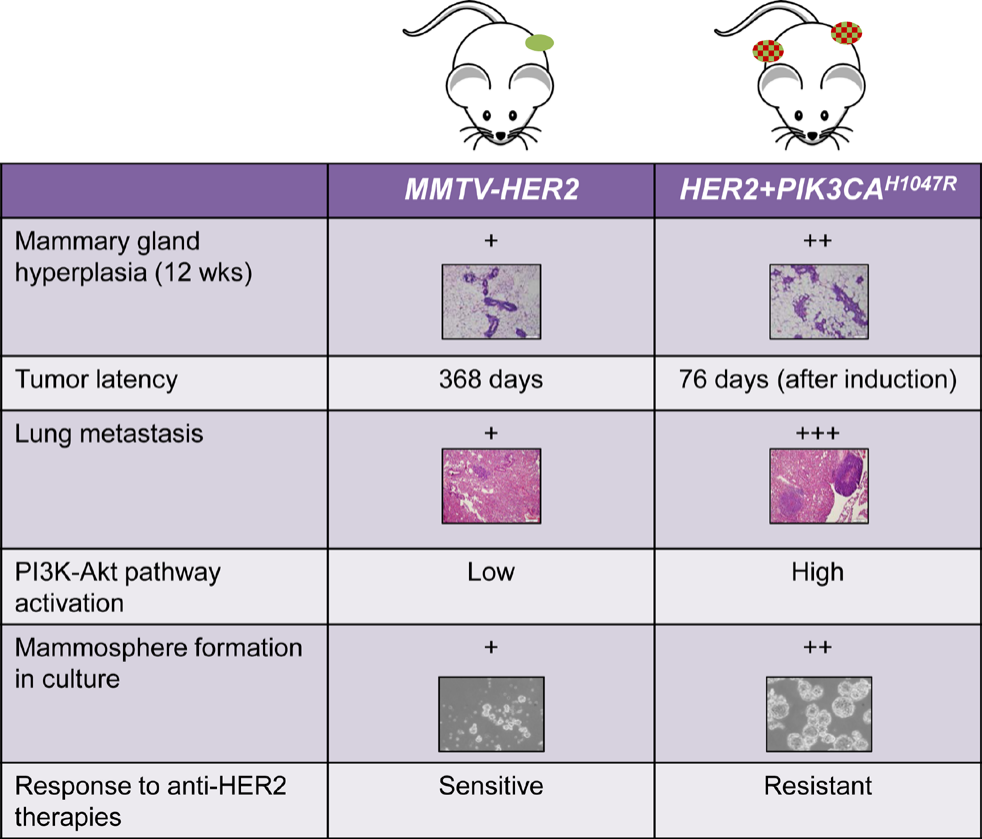
Comparison of phenotypes in MMTV-HER2 and HER2/PIK3CAH1047R mice *HER2/PIK3CA* mice developed more mammary gland hyperplasia, formed tumors faster, and formed larger and more lung metastases than tumors expressing *HER2* alone. *HER2/PIK3CA* tumor cells also formed larger mammospheres and contained higher phospho-Akt. Importantly, while *MMTV-HER2* tumors were sensitive to anti-HER2 therapies, *HER2/PIK3CA* tumors were completely resistant to both single-agent trastuzumab and combinations of HER2 inhibitors.

Approximately one in four breast cancers is driven by amplification of the receptor tyrosine kinase HER2 (ERBB2). HER2 signals through oncogenic pathways, such as the phosphatidylinositol-3-kinase (PI3K)-Akt pathway. Blockade of HER2 with FDA-approved drugs such as trastuzumab, lapatinib, and pertuzumab has changed the natural history of HER2-positive (HER2+) breast cancers and improved patient survival. However, resistance to anti-HER2 therapies represents an enormous hurdle to the eradication of this subtype of breast cancer. Activating mutations in *PIK3CA*, the gene that encodes the p110α catalytic subunit of PI3K, occur in 30-40% of HER2+ breast cancers. Several studies have found a correlation between PI3K pathway activation and resistance to anti-HER2 therapies (Esteva FJ, et al. Am J Pathol. 2010; 177:1647; Chandarlapaty S et al. Clin Cancer Res. 2012; 18:6784). However, clinical data on this topic have been inconsistent, and confirmation of a causal association between *PIK3CA* mutations and resistance to anti-HER2 therapies is lacking. To formally test this hypothesis, we generated a genetically engineered mouse model of HER2+/*PIK3CA*-mutant breast cancer [1]. In these *HER2/PIK3CA* mice, HER2 and mutant PI3K (containing the H1047R kinase domain mutation) strongly cooperated to promote mammary gland hyperplasia, tumor growth and lung metastasis (Fig. 1). In contrast to tumors expressing only *HER2, HER2/PIK3CA* tumors were completely resistant to single-agent trastuzumab and to combinations of anti-HER2 therapies. This resistance was partially reversed by combined treatment with a PI3K inhibitor currently in clinical trials.

Importantly, mutant *PIK3CA* altered the intrinsic phenotype of HER2+ tumors while increasing characteristics of cancer stem cells (CSCs) [1]. Whereas *MMTV-HER2* tumors were histologically homogeneous, expressed luminal markers, and exhibited a gene expression profile most similar to human luminal tumors, *HER2/PIK3CA* tumors were highly heterogeneous, expressed both luminal and basal markers, and exhibited a gene expression profile reminiscent of human claudin-low breast cancers, a subtype characterized by poor differentiation and elevated expression of epithelial-to-mesenchymal transition (EMT) and CSC markers [2]. In agreement, *HER2/PIK3CA* tumors expressed elevated EMT and CSC markers. Further, *HER2/PIK3CA* tumor cells more efficiently formed mammospheres in culture, a surrogate assay for tumor-initiating capacity. Finally, cells from *HER2/PIK3CA* tumors formed substantially more and larger lung metastases than cells from *HER2* tumors. These findings suggest that human HER2+ breast cancers harboring *PIK3CA* mutations may display a more virulent behavior, with greater plasticity to circumvent therapeutics. In support of this, a recent study found that human HER2+ breast cancers enriched in tumor initiating cell gene signatures contained higher PI3K pathway activity [3]. Thus, HER2+/*PIK3CA*-mutant breast cancers may benefit from treatments that target both the rapidly-dividing bulk populations and relatively quiescent tumor-initiating cancer cells.

The discrepancies in the clinical data regarding *PIK3CA* mutation status and resistance to trastuzumab could potentially be due to discordance of *PIK3CA* mutations between primary and metastatic biopsies [4], clonal heterogeneity within the tumor [5] and/or the addition of chemotherapy to HER2-targeted drugs. Further, biopsies from primary tumors could miss *PIK3CA* mutations in metastatic sites. Thus, *PIK3CA* mutational status should also be assessed in cell-free plasma tumor DNA or in metastatic sites in order to stratify patients that may require PI3K inhibitors in addition to anti-HER2 therapies. *PIK3CA* mutations should also be assessed in HER2+ tumors that recur following anti-HER2 therapy as *PIK3CA* mutations may be enriched in recurrent disease.

Clinical studies have shown that combinations of anti-HER2 therapies, such as trasuzumab + lapatinib or trastuzumab + pertuzumab, are more effective in HER2-amplified cancers than single-agent trastuzumab (Baselga et al. Lancet. 2012; 379:633). Interestingly, the CLEOPATRA study found that *PIK3CA* mutations were associated with a poorer prognosis following treatment with trastuzumab + pertuzumab + docetaxel (Baselga J et al. 2012 CTRC-AACR San Antonio Breast Cancer Symposium. San Antonio, TX). Concordant with these data, *HER2/PIK3CA* tumors were resistant to trastuzumab alone and in combination with lapatinib or pertuzumab. However, the PI3K inhibitor BKM120 in combination with anti-HER2 therapies inhibited tumor growth [1]. This suggests a causal association between *PIK3CA* mutations and resistance to HER2 inhibitors, and supports the prompt exploration of this drug combination clinically.

Despite tumor growth inhibition, BKM120 combined with two HER2 inhibitors did not completely eliminate tumors, suggesting that additional treatments will be needed to cure metastatic *PIK3CA* mutant, *HER2*-amplified breast cancers. PI3K inhibition resulted in feedback activation of HER3 and MEK-ERK signaling, which was not circumvented by HER2 inhibition. Therefore, the addition of a HER3 or MEK inhibitor may prevent feedback compensation and maximize tumor regression. *HER2/PIK3CA* tumors may also respond to the antibody-drug conjugate trastuzumab emtansine (T-DM1), a recently approved drug for HER2+ breast cancer.

In summary, our mouse model of HER2+/*PIK3CA*-mutant breast cancer provided novel insights into the pathogenesis of this disease that may be exploited therapeutically. This model will be instrumental for understanding mechanisms of acquired resistance to anti-HER2 combinations and optimizing therapeutic strategies for this subtype of breast cancer.
